# Validation of the self-compassion scale in a sample of Italian special needs teacher

**DOI:** 10.3389/fpsyg.2023.1103021

**Published:** 2023-03-02

**Authors:** Benedetta Ragni, Francesco Sulla, Giusi Antonia Toto, Pierpaolo Limone

**Affiliations:** Department of Human Studies, University of Foggia, Foggia, Apulia, Italy

**Keywords:** self-compassion, Italian validation, resilience, special needs teacher, anxiety

## Abstract

**Introduction:**

Special needs teachers deliver crucial care to their students by showing a particular attitude toward them. However, they usually face stressful situations that negatively impact their capacity to support their students, often reporting higher levels of burnout compared to teachers from mainstream education. Self-compassion has been seen to function as a protective factor against teacher stress, enhancing their resilience and coping abilities.

**Methods:**

The current study aimed to evaluate the factorial structure, reliability, and validity of the Self-compassion Scale (SCS) in a sample of Italian special needs teachers (R1). In addition, it was investigated whether the satisfactory internal reliability of the SCS is confirmed (R2). Finally, the validity of the SCS criterion was assessed, assuming that each of its subscales would be related to anxiety, measured with GAD-7, and with resilience, measured with the BRS (R3). A sample of 629 teachers was enrolled in this study and completed an online questionnaire.

**Results:**

Overall, the confirmatory factor analysis showed good or acceptable indices of fit to the data supporting the use of SCS to measure self-compassion in Italian special needs teachers.

**Discussion:**

The tool could be helpful for future research to start exploring the self-compassion dimension at school as a protective factor that may foster teachers’ and, consequently, students’ well-being.

## Introduction

According to the National Foundation for Educational Research (NFER), teachers experience greater job-related stress than other professionals. This is because teachers are required to handle several demands and responsibilities that underlie the use of social and emotional skills, such as emotional support, availability and regulation to students, and effective classroom management of students’ challenging behaviors (Jennings, 2015).

This is particularly true for special needs teachers. Research has shown that special needs teachers are a more vulnerable group than teachers from mainstream education, usually facing stressful situations that negatively impact their capacity to support their students (e.g., Brackenreed and Barnett, 2006; Jones and Youngs, 2012; Carver-Thomas and Darling-Hammond, 2017). Students with disabilities, indeed, are more likely to have behavioral difficulties and school failure due to severe behavioral and emotion regulation problems (e.g., Cibralic et al., 2019; Girgis et al., 2021). Furthermore, teachers’ negative emotions that result from students’ problematic behaviors negatively affect their perceived self-efficacy and motivation (Limone et al., 2021; Toto and Limone, 2021). According to literature, teachers perceived emotional distress is associated with higher levels of burnout (Wink et al., 2021) and worsening teachers’ and students’ behaviors and wellbeing (Sulla et al., 2019). Specifically, special needs teachers who experience burnout (as conceptualized in ICD-11; World Health Organization, 2018) suffer from emotional exhaustion (e.g., they could experience fatigue in confronting repetitive student’s assignments from their individualized program, or they could be too exhausted after work to engage in self-care actions); depersonalization (e.g., overlooking students’ needs in terms of problematic behaviors or endeavors to engage); and fewer accomplishment, perceiving that their efforts are useless or impeded by the organizational system in which they work (Ansley et al., 2016; Garwood et al., 2018; Brunsting et al., 2022).

Among teachers’ intrapersonal protective factors that could impact burnout, self-compassion has been demonstrated to reduce work-related stress levels. Scholars agree in defining self-compassion as both a trait and an acquirable ability (Dodson and Heng, 2022). Specifically, Kristine Neff refers to self-compassion in terms of “*how we relate to ourselves in instances of perceived failure, inadequacy, or personal suffering*” (Neff, 2022, p. 2). According to a recent review on the role of self-compassion in organizational settings conducted by Dodson and Heng (2022), employees’ self-compassion is significantly and positively related to higher mental and physical health functioning of employees in terms of lower levels of perceived depressive symptoms (Kotera et al., 2021), work–family conflict (Rafique et al., 2018), burnout (especially in care workers; Prudenzi et al., 2021; Schabram and Heng, 2021), stress levels (van der Meulen et al., 2021), higher sleep quality (Vaillancourt and Wasylkiw, 2019), healthier behaviors related to nutrition and physical activity (Horan and Taylor, 2018), higher levels of resilience (Franco and Christie, 2021), job satisfaction (Vaillancourt and Wasylkiw, 2019), and job performance (Reizer, 2019). Self-compassion is also related to self-concept. For example, self-compassion has been seen to function as a mediator in the relationship between self-concept and resilience (Katsumata and Mohanan, 2020); and self-compassion resulted to be a significant predictor of a specific form of self-concept which is professional self-concept (Zhou et al., 2022). Self-concept is usually measured using the Self-Concept Scale Form-5 (AF5), both in adolescents (García O. F. et al., 2018; Garcia and Serra, 2019; Queiroz et al., 2020; Fuentes et al., 2022) and adults (García et al., 2011; Martinez-Escudero et al., 2020; Villarejo et al., 2020). The scale’s dimensional structure has been tested with exploratory analyzes (Garcia and Musitu, 2009) and also with confirmatory factor analyzes (Tomás and Oliver, 2004) in different cultural contexts (e.g., García et al., 2006, 2013; Murgui et al., 2012; Garcia F. et al., 2018; Garcia and Serra, 2019; Chen et al., 2020). Furthermore, recent studies demonstrated a positive relationship between self-compassion and self-esteem (e.g., Eraydın and Karagözoğlu, 2017) and their effect on people’s wellbeing: in particular, Ding and Xu (2021) found that self-compassion moderated the relationship between attachment anxiety and self-esteem in a sample of 1,000 participants aged over 50 years in China and observed a moderated mediation effect of self-compassion in the relationship of attachment anxiety and self-esteem. In addition, the indirect effect of self-compassion was significant only between the attachment anxiety dimension and subjective wellbeing through self-esteem. This, as stated by the authors, “indicates that the mechanism of self-compassion is more complex than has been established in prior studies. The focus in most recent research related to self-compassion has been on its mediating role between adult attachment and well-being” (p. 8), so their results complement the findings in previous studies (e.g., Neff and Faso, 2015; Moreira et al., 2016), demonstrating also the moderating role of self-compassion. Self-esteem has been extensively measured with the Rosenberg Self-Esteem Scale (Rosenberg, 1965). This scale is one of the most extensively used instruments to assess the concept of global self-esteem (Raboteg-Saric and Sakic, 2014).

In particular, with regard to teachers, Chen (2022) found that self-compassion promotes teachers’ resilience, “*reflecting seemingly a transformative journey from a place of self-judgment to self-kindness, psychological isolation to psychological connectedness, and emotional rumination to emotional mindfulness*” (p. 1). Moreover, in a study on 35 preschool teachers in Northern California who had to deal with challenging students, Jennings (2015) found that self-compassion–together with mindfulness–represents important contributors to their social and emotional competence. In addition to this, self-compassion resulted in a protective factor for teachers’ stress to the extent that supports the development of teachers’ social and emotional competence, including both intrapersonal (self-awareness and self-management) and interpersonal dimensions (social awareness and relationship management). Although this might be the case also for special education teachers, who must daily face the challenging behaviors and emotional states of their students with disabilities, to date, no studies have taken into account the protective role of self-compassion in enhancing special education teachers’ wellbeing.

### The role of self-compassion in education and special education settings

Caring is a key factor for successful education (Noddings, 2018). In educational contexts, indeed, it allows the creation of a solid relationship between the teacher and the student that is characterized by receptivity and responsiveness. A good relationship between a teacher and their students has positive effects on both students’ learning processes and teachers’ self-efficacy and wellbeing (Poulou et al., 2019; Sulla and Rollo, 2023). According to Gilbert (2000), care-seeking behaviors activate the affiliative system, self-compassion, and compassion toward others. In addition to this, taking into account Fogel and colleagues’ definition of care-nurturance, “*the provision of guidance, protection, and care to foster developmental change*” (Fogel et al., 1986; p. 70), having a compassionate mindset means being supportive, understanding, kind, and helpful to others (Beaumont et al., 2022). These features clearly characterize special education contexts: special needs teachers deliver crucial care to their students by showing a particular attitude toward them (De Stasio et al., 2019, 2020).

Thus, according to these studies, a compassionate mindset and its related skills in terms of caring and self-care could represent a crucial factor in supporting intrapersonal and interpersonal dimensions of social and emotional competencies (Jennings, 2015) that teachers can use as protective tools for enhancing their wellbeing, their relationship with students, and consequently, students’ wellbeing and learning processes. Furthermore, considering that these caring and self-care skills resulted particularly salient in organizational contexts characterized by high levels of acute and chronic stress (Dodson and Heng, 2022), they could represent a protective factor for special needs teachers’ burnout onset.

Although self-care resulted essential in the special educational setting, it is understudied in educational research. The body of research on teachers’ self-compassion is recent and showed that higher self-compassion is related to teachers’ lower stress (Hwang et al., 2019) and higher teaching efficacy (e.g., Moè and Katz, 2020). However, it includes few studies (e.g., Jennings, 2015; Akpan and Saunders, 2017; Hwang et al., 2019; Moè and Katz, 2020) and none of them involving special education teachers.

### The current study

Neff (2003a) has defined self-compassion as a “*healthy form of self-acceptance, which involves being touched by one’s own suffering, along with the desire to alleviate it and treat oneself with understanding and kindness*” (p. 224). Specifically, it comprises three dimensions, each consisting of two contrasting factors: (1) self-kindness vs. self-judgment, which is the ability to be caring with oneself rather than self-critical; (2) common humanity vs. isolation, which represents the capacity to remind ourselves that suffering is natural for human beings; and (3) mindfulness vs. over-identification factor which concerns understanding and acceptance of our painful experiences without judging ourselves (Neff, 2003a; Neff et al., 2005). According to the Author these three elements are associated with and foster one another. For example, a mindful understanding of failures could reduce self-judgment; or considering them as a natural part of human beings could help to prevent judging ourselves (Barnard and Curry, 2011).

Within this theoretical framework, Neff has developed the Self-Compassion Scale (SCS; Neff, 2003a), a 26-item questionnaire composed of six dimensions: Self-Kindness, Self-Judgment, Common Humanity, Isolation, Mindfulness, and Over-Identification. In validating the scale, indeed, Neff (2003a) found that a six-factor model fitted the data better than a three-factor model for each component (self-kindness vs. self-judgment; common humanity vs. isolation; mindfulness vs. over-identification).

Several studies have demonstrated its good internal consistency, test–retest reliability, and construct validity (Barnard and Curry, 2011), including the one of Petrocchi et al. (2014) conducted in the Italian context. The scale has been resulted valid and reliable in measuring self-compassion in the general population. However, it has never been validated on teachers’ samples.

Having a reliable instrument to measure special needs teachers’ self-compassion could help researchers and clinicians obtain data that could guide interventions aimed at fostering teachers’ self-compassion as a protective factor for their stress levels and caring skills toward students. In addition to this, it could help to obtain data aimed at understanding its protective role on special needs teachers’ stress levels, which could usefully help organizational leaders working in educational settings (e.g., school headmasters, university rectors) to clearly understand what self-compassion is, and how it can promote work engagement, job performance, and wellbeing at work.

Using confirmatory factor analysis (CFA), the main aim of this study was to assess the factorial structure of the SCS in a group of special needs teachers. In particular, our research questions were:

(R1) Has the SCS’s factorial structure of the Italian validation (six-factor structure; Petrocchi et al., 2014) good fit indexes also within a sample of special needs teachers?

(R2) Is the SCS internal reliability confirmed, with a Cronbach’s alpha cut-off value not smaller than 0.70?

(R3) Is the SCS criterion validity assessed, supposing that each of its subscales will correlate with anxiety, measured with the Generalized Anxiety Disorder-7 scale (GAD-7; Spitzer et al., 2006); and with Resilience, measured with the Brief Resilience Scale (Smith et al., 2008)?

## Method

### Participants and procedure

Six hundred ninety-nine Italian special needs teachers were enrolled in this study between July and August 2022. While attending an in-person teacher training course at the University of Foggia, they completed a digitalized version of the self-report questionnaires included in this study. All participants signed informed consent, and they were secured about voluntary participation and anonymity.

The teachers come from different Italian regions and considering that this study population was a convenience sample, it may not be taken as representative of the entire population of Italian special needs teachers. This study was accepted by the Ethics Committee of the University of Foggia, Italy, and conducted in line with the Declaration of Helsinki.

### Measures

Participants completed the following questionnaires: *The Self-Compassion Scale* (SCS; Neff, 2003a,b; Petrocchi et al., 2014) assesses the extent to which people have compassionate beliefs about themselves when facing failures or challenges. Specifically, the questionnaire measured how people usually behave toward themselves in difficult times. The questionnaire is composed of 26 items rated on a five-point Likert scale ranging from 1 = “almost never” to 5 = “almost always.” Moreover, it includes six subscales: self-kindness (5 items; e.g., *I try to be loving toward myself when I’m feeling emotional pain*), self-judgment (5 items; e.g., *When times are really difficult, I tend to be tough on myself*), common humanity (4 items; e.g., *When things are going badly for me, I see the difficulties as part of life that everyone goes through*), isolation (4 items; e.g., *When I think about my inadequacies, it tends to make me feel more separate and cut off from the rest of the world*), mindfulness (4 items; e.g., *When something upsets me I try to keep my emotions in balance*), and over-identified (4 items; e.g., *When I’m feeling down I tend to obsess and fixate on everything that’s wrong*).

*The Generalized Anxiety Disorder-7 Scale* (GAD-7; Spitzer et al., 2006) represents a screening tool for detecting GAD. It is a self-report questionnaire composed of 7 items, measuring people’s anxiety symptoms during the previous 2 weeks. Items are measured on a 4-point Likert scale ranging from 0 “not at all,” to 4, “nearly every day.” Total scores vary from 0 to 21, and 5, 10, and 15 represent cut-off points for mild, moderate, and severe anxiety. In this study, Cronbach’s alpha was *α* = 0.90.

*The Brief Resilience Scale* (Smith et al., 2008) is a 6-item self-report measured on a 5-points Likert scale ranging from 1 (strongly disagree) to 5 (strongly agree), assessing psychological resilience. Higher scores indicate higher degrees of resilience. In this study, Cronbach’s alpha was *α* = 0.81.

### Data analysis

First of all, an item analysis was performed using SPSS 27 (IBM Corporation, 2020), investigating the items’ psychometric characteristics in terms of mean, standard deviation, skewness, and kurtosis. In addition to this, Mahalanobis distance (*p* < 0.001) was calculated for all scores in order to identify and skip any multivariate outliers. To explore (R1), a confirmatory factor analysis was run (CFA; Ruscio and Roche, 2012; Tabachnick and Fidell, 2013; Brown, 2015), using Mplus 8.3 (Mutheén and Mutheén, 2019). The fit indices of the model were evaluated according to systematic fit assessment procedures (Hu and Bentler, 1999; Cheung and Rensvold, 2002; McDonald and Ho, 2002; Kline, 2010) and they included (Schermelleh-Engel et al., 2003): the Chi-square test of exact fit (*χ*^2^), comparative fit index (CFI; ≥0.90; Tucker and Lewis, 1973; Bentler and Bonett, 1980; Bentler, 1990; Hu and Bentler, 1999), standardized root mean square residual (SRMR; ≤ 0.10; Tucker and Lewis, 1973; Bentler and Bonett, 1980; Bentler, 1990; Hu and Bentler, 1999), and the root mean square error of approximation [RMSEA; ≤0.08 (77,79,80,82–85)] with its 90% confidence interval (Hu and Bentler, 1999; Marsh et al., 2005).

In addition to this, according to the Italian validation of the scale (Petrocchi et al., 2014), and considering that the factorial structure of this scale is not undisputed in the literature, we also tested three alternative models. Together with the six-factor model, we assessed a model with a single higher-order self-compassion factor (Neff, 2003a), a one-factor model (Deniz et al., 2008) and a two-factor model (dividing positive and negative dimensions of self-compassion; Gilbert et al., 2011). Considering that they are non-nested models, we used three information criteria to choose the best fit: AIC, BIC, and Sample-Size Adjusted BIC. Lower values of these indices indicate a better model (Wang and Wang, 2012).

To assess (R2), the internal consistency reliability of the SCS was measured (Sexton et al., 2006; Raykov and Marcoulides, 2011; DeVellis and Thorpe, 2021). Cronbach’s alpha (α, excellent, *α* ≥ 0.9, good, *α* ≥ 0.8, acceptable, *α* ≥ 0.7, questionable, *α* ≥ 0.6, poor, *α* ≥ 0.5, and unacceptable, *α* ≤ 0.5; Cronbach, 1951) was used and reliability indices of the latent factors identified in the final model were verified including Composite Reliability (CR; cut-off values ≥0.6) and Maximal Reliability (MR; excellent, *α* ≥ 0.9, good, *α* ≥ 0.8, acceptable, *α* ≥ 0.7, questionable, *α* ≥ 0.6, poor, *α* ≥ 0.5, and unacceptable, *α* ≤ 0.5; Fornell and Larcker, 1981).

Finally, R3 was investigated, measuring the SCS construct validity through convergent and discriminant validity.

## Results

After controlling for the statistical distribution of the data (kurtosis and skewness values and Mahalanobis distance), 70 multivariate outliers were identified and deleted. Finally, 629 teachers (85% female) aged from 22 to 60 (*M* = 39.00; SD = 8.00), composed our sample.

Table 1 shows teachers’ demographic characteristics in the final sample (*N* = 629): 68% have a university degree or postgraduate specialization, and 18% have a high school diploma. Participants worked in kindergartens (7.7%), primary schools (27.8%), middle schools (30.1%), and high schools (34.4%). Overall, the items show acceptable skewness and kurtosis values (Table 2).

**Table 1:** Descriptive statistics.

Variables	*M* (SD)	*N*	%
Age (years)	39.00 (8.00)		
Gender			
*F*		533	84.70%
*M*		96	15.30%
Education			
High school		113	18%
University degree		428	68%
Postgraduate specialization		88	14%
Working school level			
Kindergarten		30	7.70%
Primary school		109	27.80%
Middle school		118	30.10%
High school		135	34.40%

**Table 2:** Skewness and kurtosis values.

	Skewness	SE	Kurtosis	SE
Self-kindness				
SC5	−0.082	0.097	−0.558	0.195
SC12	−0.191	0.097	−0.714	0.195
SC19	−0.005	0.097	−0.523	0.195
SC23	−0.149	0.097	−0.373	0.195
SC26	−0.069	0.097	−0.382	0.195
Self-judgment				
SC1	0.259	0.097	−0.346	0.195
SC8	−0.112	0.097	−0.88	0.195
SC11	−0.26	0.097	−0.63	0.195
SC16	−0.762	0.097	−0.323	0.195
SC21	−0.175	0.097	−0.804	0.195
Common humanity				
SC3	−0.518	0.097	−0.42	0.195
SC7	−0.266	0.097	−0.842	0.195
SC10	−0.229	0.097	−0.715	0.195
SC15	−0.487	0.097	−0.262	0.195
Isolation				
SC4	−0.55	0.097	−0.735	0.195
SC13	−0.637	0.097	−0.551	0.195
SC18	−0.692	0.097	−0.493	0.195
SC25	−0.083	0.097	−1.054	0.195
Mindfulness				
SC9	−0.583	0.097	−0.394	0.195
SC14	−0.469	0.097	−0.326	0.195
SC17	−0.429	0.097	−0.383	0.195
SC22	−0.1	0.097	−0.616	0.195
Over-Identified				
SC2	−0.218	0.097	−0.964	0.195
SC6	−0.024	0.097	−0.901	0.195
SC20	−0.057	0.097	−0.584	0.195
SC24	−0.187	0.097	−0.926	0.195

SE, Standard error.

### Factorial validity

Table 3 reports the fit indices of the four tested models. The six-factor model showed the lowest AIC, BIC, and Sample-Size Adjust BIC, and, therefore, the best fit (Wang and Wang, 2012). Analyzing the factor loadings of this model, we found that item 1 had a factor loading value < of |0.3|, and we deleted it (Brown, 2015). In addition to this, to improve the fit of the model, we checked modification indices. According to Jöreskog and Sörbom (1993), we identified the largest modification index, we estimated it, and we maintained it in the model only if the modified parameter could be interpreted substantively according to our theoretical framework. At the end of the process, we determined that covariances between the errors of three couples of items could be included in the final model (item11 and item8 *r* = 0.417, *p = 0*.000; item10 and item15 *r* = 0.283, *p* = 0.000; item5 and item19 *r* = 0.279, *p* = 0.000).

**Table 3:** Factorial validity.

Model	RMSEA 90% CI										
	*χ*2	*p*	df	CFI	SRMR	RMSEA	Lower	Upper	AIC	BIC	Sample-Adj BIC
One-factor	1942.057	0.00	299	0.695	0.089	0.093	0.090	0.097	46932.155	47278.797	47031.157
Two-factor	1411.871	0.00	298	0.793	0.780	0.077	0.073	0.081	46293.359	46644.445	46393.63
High-order factor	1,675,393	0.00	298	0,744	0,114	0,086	0,020	0,090	46606.704	46957.79	46706.974
Six-factor	1275.445	0.00	284	0.816	0.080	0.074	0.070	0.079	46158.43	46571.734	46276.47
Six-factor final model*	1056.658	0.00	257	0.847	0.073	0.070	0.066	0.075	44186.851	44600.155	44304.892

*item1 deleted; covariances added between: item11 and item8, item10 and item15, item5 and item19.

The confirmatory factor analysis (R1) of the six-factor final model showed acceptable indices of fit to our data (MLM *χ*^2^(257) = 1056.658, *p* < 0.001, RMSEA = 0.070, 90% CI [0.066, 0.075], CFI = 0.847, and SRMR = 0.073; Table 3). Factor loadings are reported in Table 4. All the six factors showed significant covariances: Self-Judgment with Self-Kindness (*r* = 0.644, *p* = 0.000); Common Humanity with Self-Judgment (*r* = 0.519, *p* = 0.000) and Self-Kindness (*r* = 0.878, *p* = 0.000); Isolation with Common Humanity (*r* = 0.630, *p* = 0.000), Self-Judgment (*r* = 0.943, *p* = 0.000), and Self-Kindness (*r* = 0.676, *p* = 0.000); Mindfulness with Isolation (*r* = 0.680, *p* = 0.000), Common Humanity (*r* = 0.866, *p* = 0.000), Self-Judgment (*r* = 0.560, *p* = 0.000), and Self-Kindness (*r* = 0.948, *p* = 0.000); and Over-Identified with Mindfulness (*r* = 0.698, *p* = 0.000), Isolation (*r* = 0.965, *p* = 0.000), Common Humanity (*r* = 0.573, *p* = 0.000), Self-Judgment (*r* = 0.907, *p* = 0.000), and Self-Kindness (*r* = 0.698, *p* = 0.000).

**Table 4:** Factor loadings and reliability.

	Estimate	SE	Cronbach’s α	Omega	CR	MR
Self-kindness			0.84	0.85	0.757	0.761
Item 5	0.638	0.028				
Item 12	0.605	0.029				
Item 19	0.626	0.028				
Item 23	0.547	0.031				
Item 26	0.679	0.025				
Self-judgment			0.81	0.82	0.644	0.664
Item 8	0.431	0.036				
Item 11	0.552	0.033				
Item 16	0.678	0.027				
Item 21	0.562	0.034				
Common humanity			0.70	0.85	0.711	0.716
Item 3	0.636	0.031				
Item 7	0.668	0.028				
Item 10	0.544	0.032				
Item 15	0.618	0.029				
Isolation			0.81	0.81	0.673	0.710
Item 4	0.383	0.038				
Item 13	0.716	0.026				
Item 18	0.664	0.025				
Item 25	0.552	0.030				
Mindfulness			0.78	0.78	0.796	0.799
Item 9	0.662	0.023				
Item 14	0.670	0.024				
Item 17	0.753	0.021				
Item 22	0.723	0.022				
Over-Identified			0.77	0.77	0.741	0.746
Item 2	0.586	0.027				
Item 6	0.708	0.023				
Item 20	0.650	0.029				
Item 24	0.636	0.031				

CR, Composite Reliability; MR, Maximal Reliability.

### Reliability

Table 4 shows the internal consistency (R2) of each SCS factor. Cronbach’s α showed values between acceptable and good for all subscales of the SCS. Specifically, we observed α = 0.84 for the Self-kindness subscale, *α* = 0.83 for the Self-Judgment subscale, *α* = 0.70 for the Common Humanity subscale, *α* = 0.81 for the Isolation subscale, *α* = 0.78 for the Mindfulness subscale, and *α* = 0.77 for the Over identified subscale. With regard to CR and MR, all the subscales presented values between acceptable and good.

### Construct validity

To investigate SCS construct validity, we performed Pearson correlations among all scales (R3; see Table 5). Regarding the relationships between the dimensions of SCS and GAD-7 (Anxiety), the GAD-7 Total Scale was positively correlated with the negative dimensions of the SCS: Self-Judgment (*r* = 0.496, *p* ≤ 0.01), Isolation (*r* = 0.520, *p* ≤ 0.01), and Over Identified Subscale (*r* = 0.546, *p* ≤ 0.01). Furthermore, the GAD-7 Total Scale was also negatively correlated with the positive dimensions of the SCS: Self-Kindness (*r* = −0.341, *p* ≤ 0.01) and Mindfulness (*r* = −0.294, *p* ≤ 0.01). Regarding the Brief Resilience Scale, the analysis highlighted significant negative correlations with the negative dimensions of the SCS: Self-Judgment (*r* = −0.460, *p* ≤ 0.01), Isolation (*r* = −0.492, *p* ≤ 0.01), and Over Identified Subscale (*r* = −0.558, *p* ≤ 0.01). In addition to this, Brief Resilience Scale was positively correlated with the positive dimensions of the SCS: Self-Kindness (*r* = 0.341, *p* ≤ 0.01), Common Humanity (*r* = −0.089, *p* ≤ 0.01), and Mindfulness (*r* = 0.426, *p* ≤ 0.01).

**Table 5:** Construct validity.

	Self-kindness	Self-judgment	Common humanity	Isolation	Mindfulness	Over identified
Anxiety (GAD-7)	−0.341^**^	0.496^**^	−0,06	0.520^**^	−0.294^**^	0.546^**^
Resilience (BRS)	0.341^**^	−0.460^**^	0.089^*^	−0.492^**^	0.426^**^	−0.558^**^
Self-kindness	-					
Self-judgment	−0.551**	-				
Common humanity	0.404**	−0.094*	-			
Isolation	−0.442**	0.714**	−0.142**	-		
Mindfulness	0.678**	−0.435**	0.442**	−0.498**	-	
Over identified	−0.449**	0.722**	−0.093*	0.773**	−0.497**	-

*p < 0.05; **p < 0.01.

## Discussion

The main aim of the current study was to evaluate the factorial structure, reliability, and validity of the SCS (Neff, 2003a,b) in an Italian sample of special education teachers. Although there is an adaptation of the SCS in Italian samples, to the best of our knowledge, there are none with teachers’ samples.

Overall, findings from CFAs showed that a six-factor model, as validated in the development and validation study by Neff (2003a) and in the Italian adaptation by Petrocchi et al. (2014), provided a good fit to the data (R1). The internal consistency of the six dimensions was high and comparable to those obtained in the two aforementioned studies (R2). All the correlations among the six subscales were in the expected direction. As in the Italian study validation, the Common Humanity subscale presented nonsignificant or weaker correlations with the negative SCS dimensions (Self-Judgment, Isolation, and Over-Identification), and with anxiety and resilience levels. The common humanity dimension estimates the feeling of affinity with others in terms of weakness and imperfections, and in our Italian sample of special needs teachers, it is poorly related to the absence of self-criticism and resilience. As explained in the Italian validation study (Petrocchi et al., 2014), it could be possible that the recognition of being limited and imperfect may lead people to judge themselves because “*they should not be suffering so much*” or “*they should get over it*.”

The subscales also demonstrated good construct validity (R3). The correlations among the six factors and the other measured variables were in the hypothesized direction. Specifically, GAD scores were positively correlated with the negative factors of the SCS (Self-Judgment, Isolation, Over Identified Subscale) and negatively correlated with the positive ones (Self-Kindness, Mindfulness). Similarly, resilience scores resulted be negatively related to the negative subscales of the SCS, and positively to the positive factors. These results indicate that teachers with high scores in the negative subscales are self-critical and reported higher levels of generalized anxiety and lower levels of resilience.

On the contrary, Italian special needs teachers that reported higher levels of self-compassion also encountered lower levels of anxiety and higher levels of resilience. According to the literature, self-compassion has been demonstrated to be a protective factor for the onset and maintenance of mental illnesses (Egan et al., 2021), and psychological resilience results related to lower levels of stress and self-compassion levels (Kemper et al., 2015; Kotera et al., 2021). Furthermore, people declaring higher levels of self-compassion were also more resilient when encountering challenging adversity and failures (Neff et al., 2007; Tiwari et al., 2020). Thus, we may hypothesize that self-compassion could also be a key factor in reducing teachers’ negative stress and mental health consequences.

## Conclusion

The current study supported the use of SCS to measure self-compassion in Italian special needs teachers. This scale could be useful for future research to start exploring the self-compassion dimension at school as a protective factor that could foster teachers’ social and emotional competencies, their relationship with students, their wellbeing, and, consequently, students’ wellbeing and learning processes. In addition to this, cross-sectional and longitudinal studies aimed at examining the role of teachers’ self-compassion in reducing stress, burnout, and technostress or in enhancing wellbeing and work engagement should be conducted to observe how and if the role of self-compassion changes when tested together with other protective and risk factors (e.g., resilience, anxiety, support received from colleagues, self-efficacy). These data could usefully inform organizational leaders working in educational settings (e.g., school headmasters, university rectors) to clearly understand what self-compassion is, and how it can promote work engagement, job performance, and wellbeing at work. Furthermore, SCS could represent a useful instrument to inform clinical interventions aimed at fostering teachers’ individual protective factors (e.g., self-compassion, mindfulness, resilience). Finally, empirical research in different countries might also be needed in order to examine the cross-cultural stability of the scale’s factorial structure and advance our understanding of the self-compassion dimension in teachers’ wellbeing.

## Data availability statement

The data presented in this study are available on request from the corresponding author. The data are not publicly available due to privacy reasons.

## Ethics statement

The study was reviewed and approved by Ethics Committee of the University of Foggia. The participants provided their written informed consent to participate in this study.

## Author contributions

BR, FS, GT, and PL designed and carried out the study. BR and GAT collected the data and performed statistical analysis. BR, FS, GT, and PL contributed to the analysis of the results and to the writing of the manuscript. GT and PL supervised the study design and the manuscript draft. All authors contributed to the article and approved the submitted version.

## Conflict of interest

The authors declare that the research was conducted in the absence of any commercial or financial relationships that could be construed as a potential conflict of interest.

## Publisher’s note

All claims expressed in this article are solely those of the authors and do not necessarily represent those of their affiliated organizations, or those of the publisher, the editors and the reviewers. Any product that may be evaluated in this article, or claim that may be made by its manufacturer, is not guaranteed or endorsed by the publisher.
